# Supplementary material for: Human OAT1, OAT3, OAT4 and OATP1A2 Facilitate the Renal Accumulation of Ochratoxin A
Source: Pharmaceutics. 2025 Nov 16;17(11):1474. doi: 10.3390/pharmaceutics17111474 (PMC12655426; doi:10.3390/pharmaceutics17111474)
Supplement: Supplementary file 1 [file pharmaceutics-17-01474-s001.zip › pharmaceutics-3944687-supplementary.pdf]

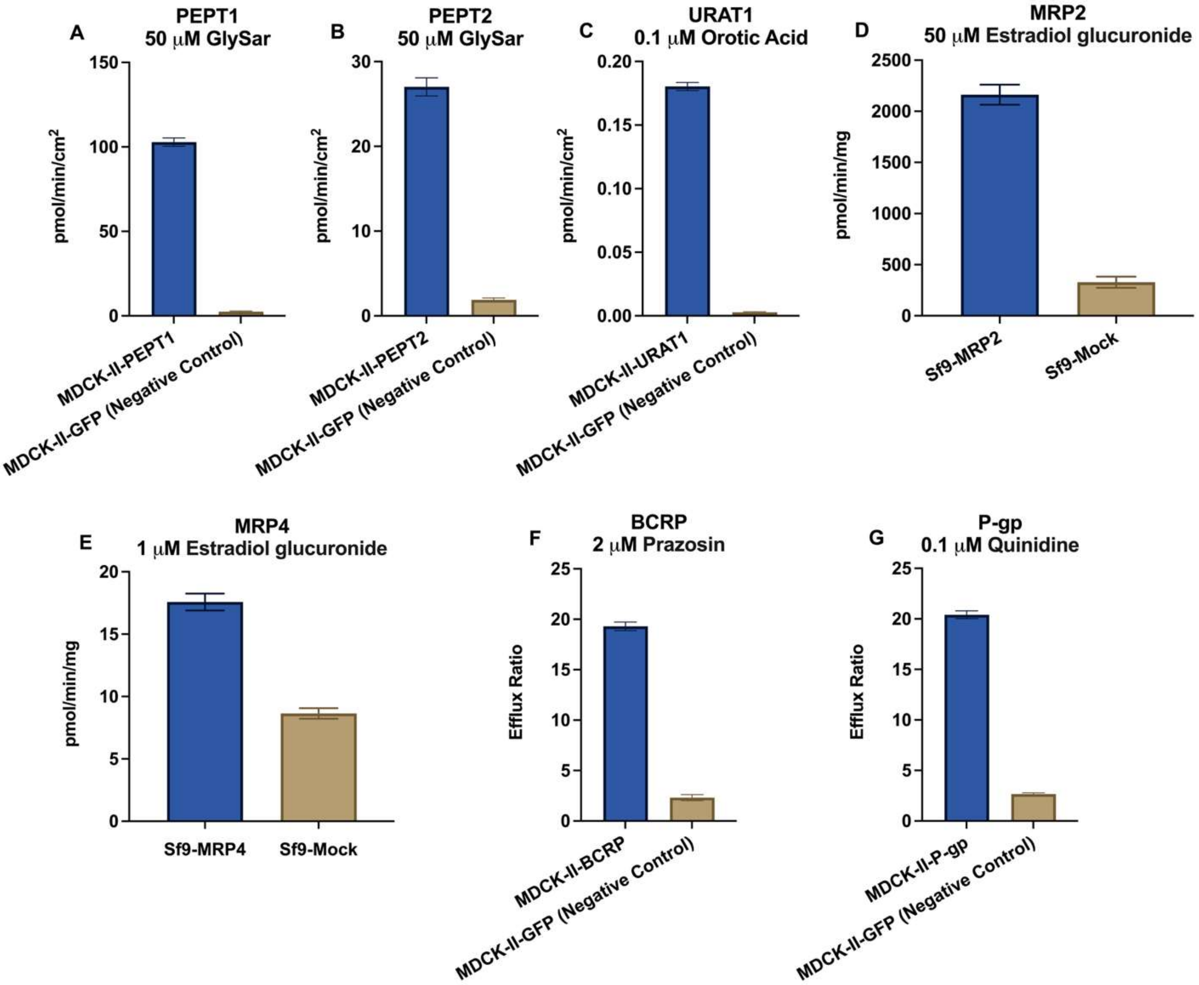

Supplemental Figure S1. Verification of functional activity in transporter-overexpressing cell lines and vesicles. For PEPT1 (A), PEPT2 (B), and URAT1 (C), transporters are expressed in MDCK-II cells cultured on transwell inserts. Data are mean ± S.D. of intracellular substrate amount (pmol substrate/incubation time/culture area) after 5-minute incubations. GFP-transfected MDCK-II cells are included as negative controls. For MRP2 (D) and MRP4 (E), transporters are expressed in Sf9 membrane vesicles. Data are mean ± S.D. of intracellular substrate amount (pmol substrate/incubation time/total protein amount) after 5-minute incubations. Mock-transfected-Sf9 membrane vesicles are included as negative controls. For BCRP (F) and P-gp (G), transporters are expressed in MDCK-II cells cultured on transwell inserts. Data are mean efflux ratios ± S.D. after 60-minute incubations. GFP-transfected MDCK-II cells are included as negative controls. Probe substrates for each transporter used are listed in the figure title. These data demonstrate robust activity of transporters in the systems used in this study.

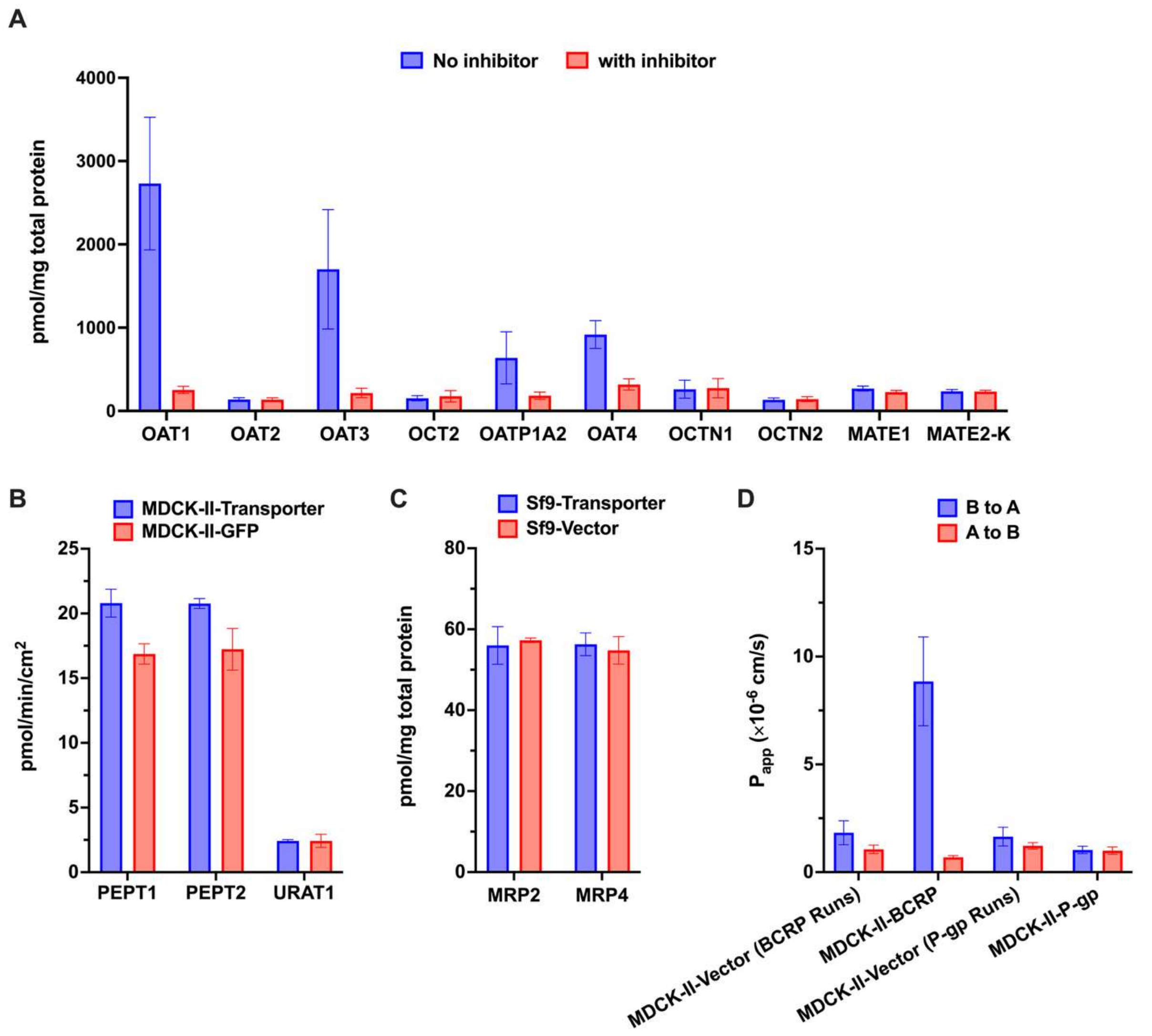

Supplemental Figure S2. Uptake of ochratoxin A (OTA) in transporter-expressing cell lines and vesicles relative to their respective negative controls. (A) Protein-normalized OTA uptake (pmol/mg of total protein) in transporter-overexpressing mammalian cells (OCTN2 overexpressed in CHO cells; other transporters overexpressed in HEK293 cells) in the absence and presence of corresponding inhibitors (i.e., 200 μM probenecid for OAT1/3, 200 μM bromsulphthalein for OAT2/4 and OATP1A2, 200 μM pyrimethamine for OCT2 and MATE1/2-K, 1 mM ergothioneine for OCTN1, 200 μM verapamil for OCTN2). (B) Protein-normalized OTA uptake in MDCK-II cells transiently-transfected with GFP (negative control), PEPT1, PEPT2, and URAT1. (C) Protein-normalized ATP-mediated OTA accumulation in control Sf9 membrane vesicles (negative control) and Sf9 membrane vesicles overexpressing MRP2 and MRP4. (D) Apparent permeability of OTA in vector-transfected MDCK-II cells (negative control), and BCRP- and P-gp-overexpressing MDCK-II cells. Data are mean ± S.D. for three independent experiments, each in technical triplicate.

Supplemental Table S1. LC-MS/MS conditions for the quantification of OTA

Ionization Mode	ESI+
**Desolvation (°C)**	350
**Desolvation Gas Flow (L/hr)**	1000
**Cone Voltage**	25
**Collision Gas Flow (L/hr)**	150
**Internal Standard (IS) (ochratoxin A-$^{13}C_{20}$)**	100 nM
**MRM Mass transition (OTA)**	m/z [M+H](+) 404.23 > 239.12
**MRM Mass transition (OTA-IS)**	m/z [M+H](+) 424.3 > 250.17
**Column**	Waters Acquity UPLC BEH Shield RP18, 1.7 um, 2.1x150 mm
**Mobile Phase A**	0.1% Formic acid in water
**Mobile Phase B**	0.1% Formic acid in acetonitrile
**Column Temperature (°C)**	55
**Flow rate (mL/min)**	0.3
**LC Gradient (%B, per minute)**	0: 30%; 1: 30%; 4: 100%; 5: 100%; 5.1: 30%; 8: 30%
